# The Healthcare Needs of Children With Down Syndrome in the First Year of Life: An Analysis of the EUROlinkCAT Data Linkage Study

**DOI:** 10.1111/ppe.13176

**Published:** 2025-02-06

**Authors:** Sarah E. Seaton, Judith Rankin, Clara Cavero‐Carbonell, Ester Garne, Mika Gissler, Maria Loane, Amanda J. Neville, Michele Santoro, Joachim Tan, David Tucker, Joan K. Morris

**Affiliations:** ^1^ Department of Population Health Sciences University of Leicester Leicester UK; ^2^ Population Health Sciences Institute Newcastle University Newcastle UK; ^3^ Rare Diseases Research Unit Foundation for the Promotion of Health and Biomedical Research in the Valencian Region Valencia Spain; ^4^ Department of Paediatrics and Adolescent Medicine, Lillebaelt Hospital University Hospital of Southern Denmark Kolding Denmark; ^5^ Department of Data and Analytics, THL Finnish Institute for Health and Welfare Helsinki Finland; ^6^ Academic Primary Health Care Centre, Region Stockholm Stockholm Sweden; ^7^ Department of Molecular Medicine and Surgery Karolinska Institutet Stockholm Sweden; ^8^ Institute of Nursing and Health Research Ulster University Belfast UK; ^9^ IMER Registry Centre for Clinical and Epidemiological Research University of Ferrara and Azienda Ospedaliero Universitario di Ferrara Ferrara Italy; ^10^ Unit of Epidemiology of Rare Diseases and Congenital Anomalies, Clinical Physiology, National Council Pisa Italy; ^11^ NIHR Gosh BRC UCL Great Ormond Street Institute of Child Health London UK; ^12^ Research, Data & Digital Directorate Public Health Wales Swansea UK; ^13^ School of Health & Medical Sciences, City St George's University of London London UK

**Keywords:** congenital anomalies, Down syndrome, epidemiology, intensive care

## Abstract

**Background:**

Globally, Down syndrome is the most common chromosomal anomaly, often co‐occurring with cardiac or gastrointestinal anomalies. There is a lack of robust data on specific healthcare needs of children with Down syndrome compared to children with other major congenital anomalies.

**Objectives:**

To quantify the healthcare needs of children with Down syndrome in the first year of life compared to children with major congenital anomalies in a large population‐based cohort across Europe.

**Methods:**

The EUROlinkCAT study was a multicentre data linkage study between congenital anomaly registries in Europe and hospital and mortality databases. Children born between 1st January 1997 and 31st December 2014 were included. Summary statistics were used to compare differences between children (those with Down syndrome compared to all major anomalies) and regions. Random‐effects meta‐analysis was used to pool results related to survival, need for intensive care and ventilation support.

**Results:**

A total of 3554 children were born with Down syndrome out of 89,081 children with major congenital anomalies. The pooled 1‐year survival was 95.4%. In every region, > 80% of children with Down syndrome had a hospital admission excluding the birth admission. Hospital length of stay in the first year was higher for children with Down syndrome compared to those with all anomalies (median: 14 versus 7 days). Despite having similar need for ventilation support (9.7% vs. 8.4%), children with Down syndrome had higher rates of intensive care admission than all children with anomalies (24.8% vs. 13.0%).

**Conclusions:**

There is a high need for hospital care for children born with Down syndrome in the first year of life. Future work should continue to explore the long‐term prognosis for children with Down syndrome to ensure their care needs are met.

## 1 Background

Congenital anomalies (conditions present at birth) are a leading cause of child mortality and morbidity globally [1]. Every year around 130,000 children are born in Europe with a major congenital anomaly [2] and they account for around 28% of deaths in the first year of life [3]. Children with congenital anomalies have greater healthcare needs, for example, they are known to spend 8.8 times longer in hospital in Europe over the first year of life (18 days vs. 2 days) than children without congenital anomalies [4].

As of 2015, there were an estimated 419,000 people with Down syndrome (Trisomy 21) living in Europe [5]. Around half of children with Down syndrome also have a cardiac anomaly and around one in 10 have gastrointestinal atresia [6]. For children with Down syndrome, survival to age 10 years is highest when they do not have an associated cardiac or digestive system anomaly [7]. The condition is also associated with increased risk of other health conditions which may require hospital admissions, for example, reduced immunity to respiratory infections [8]. Both the total and live‐birth prevalence of Down syndrome have increased over time in Europe due to increasing maternal age [9].

Children with Down syndrome have higher rates of hospitalisation and readmission, and more of their admissions are emergencies compared to children without Down syndrome [10]. Furthermore, their hospital admission rates have increased over time [10], although it is unclear if these are necessary hospital admissions. Previous work has shown that children with Down syndrome's first admission to hospital are when they are younger and last longer than children without Down syndrome  [11]. Of children admitted to intensive care, those with Down syndrome have a lower disease severity on admission but more of them require organ support than expected given their severity of illness [12]. However, much of the previous research has been from a single country or region and therefore was limited to small numbers of children.

This study aims to explore the healthcare needs of children born with Down syndrome in the first year of life, focussing on length of hospital stay, hospital admissions, surgery and intensive care and the presence of additional cardiac and gastrointestinal anomalies using data collected from a large population‐based cohort across Europe.

## 2 Methods

### 2.1 Setting

The ‘Establishing a linked European Cohort of Children with Congenital Anomalies’ (EUROlinkCAT) project was a multicentre retrospective data‐linkage cohort study. EUROlinkCAT collated data from the European Network of Congenital Anomaly Registries (EUROCAT) who were supported to link their data on children with congenital anomalies to their hospital, prescription, education and mortality information. Not all registries that were part of EUROCAT participated in the EUROlinkCAT study. All those registries that were able to link their data on births to mortality and hospital discharges were included in this paper. The overall aim of the study was to examine mortality and morbidity outcomes of children born with congenital anomalies in the first 10 years of their lives. Information on individual children was not shared with the EUROlinkCAT team; rather, statistical analyses were run by each individual registry, and the resulting aggregated data and analytical results on specific topics of interest were then stored in a central results repository in Ulster University [2]. These aggregated results were transferred to the study team to allow for the analyses in this paper. Definitions of length of stay, obstetric stay, and surgery in the study have been published previously [13, 14].

In this study, we used the data on children born with a major congenital anomaly from 1st January 1997 to 31st December 2014 and followed up to 31st December 2015 from eight European regions: Tuscany (Italy); Emilia Romagna (Italy); Finland; Thames Valley (UK); Wessex (UK); East Midlands and South Yorkshire (EMSY, UK); Wales and Valencian Region (Spain). We focussed on outcomes and care provided in the first year of life.

Data are collected on all children with a confirmed diagnosis of a major congenital anomaly in the regions covered by EUROlinkCAT. In this paper, we focus on the subgroup of children who have a diagnosis of Down syndrome occurring with or without cardiac/gastrointestinal anomalies. Information was collated by EUROlinkCAT on children with Down syndrome's need for hospital care, including specific procedures such as surgery, and we focus on the first year after birth.

EUROlinkCAT defines a major congenital anomaly using the definition by the World Health Organisation [15], that is, children living with major structural changes or chromosomal abnormalities that result in significant medical, social or cosmetic changes and typically require medical intervention, for example, spina bifida, anencephaly and orofacial clefts. Children with minor anomalies, for example, single palmar crease and clinodactyly (curvature of the finger), were excluded from EUROlinkCAT [16].

### 2.2 Statistical Analysis

We compared the Kaplan–Meier 1‐year survival of children with Down syndrome across the different regions, obtaining an overall estimate using a random‐effects meta‐analysis [17] as we had summary data for each region. The Kaplan–Meier approach was used to account for censoring due to the study period ending 31st December 2014, meaning some children never reached their fifth birthday, although the censoring is negligible in this work as we have restricted the analysis to the first year. Therefore, in this work, the Kaplan–Meier estimates are similar to observed proportions. We present findings using a forest plot. We also report the observed 1‐year survival for children with Down syndrome and/or congenital heart disease (CHD) and/or gastrointestinal anomalies. The group of ‘all children with a major congenital anomaly’ includes those with Down syndrome.

We compared selected clinical needs of all children with a major congenital anomaly to only those children with Down syndrome using summary statistics (total and percentages estimated using Kaplan–Meier to account for censoring). We compared the percentage of children who were admitted to hospital in the first year of life, need for surgery, therefore, and their length of stay in hospital outside of the birth admission. The total percentages are estimated from a random‐effects meta‐analysis to allow for variation between countries.

To estimate pooled estimates of median length of stay, we used quantile estimation methods [18]. These methods use the reported median and quartiles for each registry to select an underlying parametric distribution based on the best fit of normal, log‐normal, gamma and Weibull distributions. The asymptotic variance of the median can then be calculated, and a random‐effects meta‐analysis performed [19]. We considered this for all anomalies; Down syndrome and Down syndrome with/without the additional presence of CHD.

Data on intensive care and ventilation were available for a smaller subset of data due to changes in data collection procedures over time in the hospital databases. All analyses for these outcomes were therefore performed on subsets of data during which these outcomes were reported. We considered the percentage of children (by all anomalies and those with Down syndrome) who required admission to intensive care or required ventilation support, again pooling overall estimates using a random‐effects meta‐analysis and presenting using forest plots.

### 2.3 Missing Data

We were unable to impute for missing data as we did not have individual‐level data, and when the data were missing (e.g., for intensive care data), this was at a registry level and therefore systematically missing.

### 2.4 Ethics Approval

All registries obtained ethical and other permissions for the data linkage according to their national legislations. Ulster University obtained ethics permission for the Central Results Repository, which hosts the EUROlinkCAT Central Repository, on 15th September 2017 (Institute of Nursing and Health Research Ethics Filter Committee, Number FCNUR‐17‐000).

## 3 Results

We obtained aggregated data related to 89,081 liveborn children with congenital anomalies born from 1st January 1997 to 31st December 2014 across eight European regions covering a birth population of over 3.6 million. The largest population of children were from Finland (*n* = 38,324, Table 1). In total, 3554 (4.0%) children with a diagnosis of a congenital anomaly had Down syndrome.

**TABLE 1:** Number of children aged < 1 year by anomaly status, outcome and admission to hospital.

Registry (years)	Birth population covered (1000 s)^a^	All children with a major congenital anomaly (including Down syndrome)		All children with Down syndrome		Children with Down syndrome and CHD present		Children with Down syndrome and no CHD	
		Hospital admission (total births)	%, (95% CI)^b^	Hospital admission (total births)	%, (95% CI)^b^	Hospital admission (total births)	%, (95% CI)^b^	Hospital admission (total births)	%, (95% CI)^b^
Tuscany, Italy (2005–2014)	300	3938 (4225)	93.2, (92.4, 94.0)	143 (148)	96.6, (92.8, 98.7)	52 (54)	96.3, (88.7, 99.3)	91 (94)	96.8, (91.7, 99.1)
Emilia Romagna, Italy (2008–2014)	282	5051 (5381)	93.9, (93.2, 94.5)	202 (206)	98.1, (95.4, 99.4)	78 (79)	98.7, (93.9, 99.9)	124 (127)	97.6, (93.8, 99.4)
Finland (1997–2014)	953	23,156 (38324)	60.7, (60.3, 61.2)	1001 (1205)	83.4, (81.2, 85.4)	649 (722)	90.2, (87.9, 92.2)	352 (483)	73.2, (69.2, 77.1)
Wales (1998–2014)	559	12,429 (17448)	71.9 (71.3, 72.6)	460 (562)	82.1, (78.9, 85.2)	302 (349)	86.6, (82.8, 89.9)	158 (213)	74.8 (68.8, 80.5)
Thames Valley, UK (2005–2013)	270	3452 (3845)	92.1, (91.2, 93.0)	237 (262)	92.9, (89.0, 95.8)	79 (80)	100.0^c^	158 (182)	89.2, (83.8, 93.5)
Wessex, UK (2004–2014)	325	3797 (4320)	89.5, (88.5, 90.4)	304 (334)	92.7, (89.4, 95.3)	85 (91)	95.7, (89.4, 98.8)	219 (243)	91.6, (87.5, 94.8)
EMSY, UK (2003–2012)	717	10,116 (11278)	91.8, (91.2, 92.3)	633 (682)	94.2, (90.6, 94.6)	233 (243)	98.3, (95.5, 99.5)	400 (439)	92.3, (89.4, 94.6)
Valencian Region, Spain (2010–2014)	228	4112 (4260)	96.5, (96.0, 97.0)	152 (155)	98.1, (94.9, 99.5)	101 (101)	100.0^c^	51 (54)	94.4, (86.1, 98.5)
Total	3634	66,051 (89081)	86.2, (75.6, 96.7)	3132 (3554)	92.3, (87.9, 96.6)	1579 (1719)	94.3, (90.1, 98.4)	1553 (1835)	88.9 (82.8, 94.9)

Abbreviations: CHD, congenital heart disease; CI, confidence interval; EMSY, East Midlands and South Yorkshire; UK, United Kingdom.

^**a**^ Extracted from the EUROCAT website (https://eu‐rd‐platform.jrc.ec.europa.eu/eurocat/eurocat‐data/prevalence_en), accessed on September 30, 2021.

^b^ Kaplan–Meier estimates to account for censoring.

^c^ Not calculated and excluded from pooled estimate.

**FIGURE 1:**
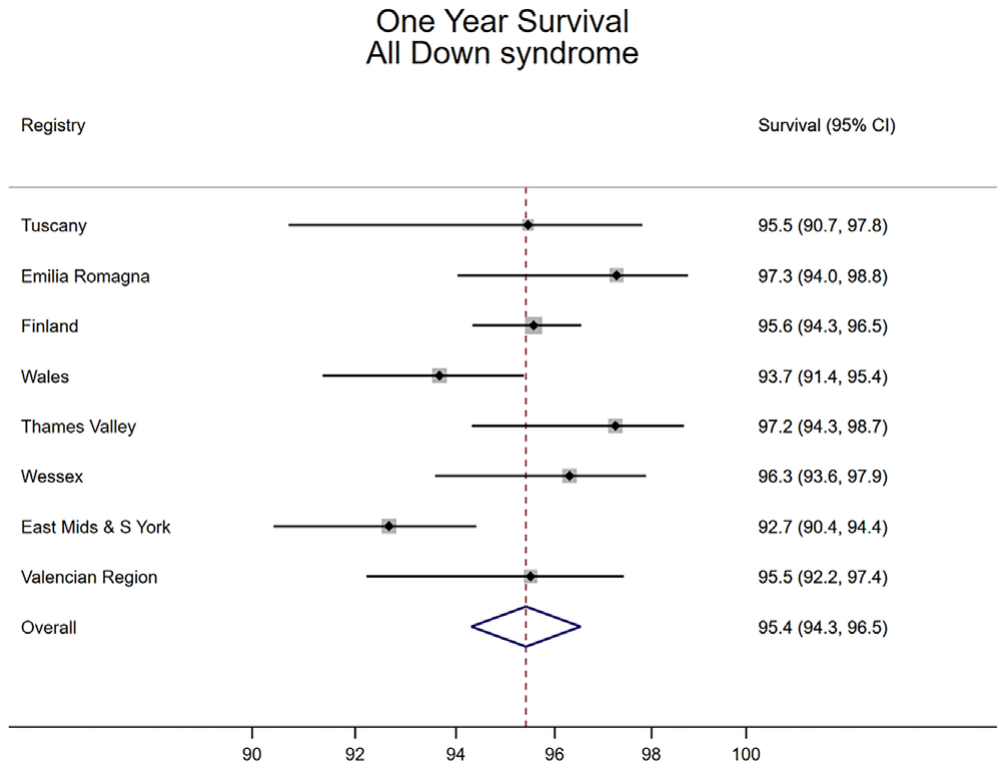
One year survival of children with Down syndrome.

For children with Down syndrome, survival at 1 year was high across all European regions, ranging from 92.7% (EMSY) to 97.3% (Emilia Romagna) (Figure 1). We obtained a pooled estimate of overall survival of 95.4% (95% CI 94.3, 96.5) across all regions (Figure 1). Survival was lower for children with associated anomalies, for example, in children with CHD and a gastrointestinal anomaly pooled survival was 88.9% (95% CI 84.2, 93.6) (Table S1).

### 3.1 Hospitalisation and Length of Stay

For children with all anomalies, the percentage of children admitted to hospital (outside of the birth admission) ranged from 60.7% (Finland) to 96.5% (Valencian Region) (Table 1). However, this was higher for children with Down syndrome, with all regions reporting hospital admission percentages of over 80% (Table 1).

Children with Down syndrome had a longer hospital length of stay in the first year of life (Table 2) than children with all anomalies across all regions (Table 2). The median lengths of stay varied from 3 days longer (Wessex) to 12 days longer (Tuscany and Finland). There were also notable regional differences between children with Down syndrome and CHD having longer stays than those children with Down syndrome without CHD. The total median length of stay in the first year for these children ranged from 15 days (Wales) to 33 days (Tuscany) (Table 2).

**TABLE 2:** Length of hospital stay in the first year of life (excluding initial birth admission) (days).

	Length of stay in hospital for children with all anomalies, median (25th, 75th)	Length of stay in hospital for children with Down syndrome, median (25th, 75th)	Length of stay in hospital for children with Down syndrome and CHD present, median (25th, 75th)	Length of stay in hospital for children with Down syndrome and no CHD, median (25th, 75th)
Tuscany, Italy	7 (3, 21)	19 (7, 43)	33 (16, 79)	11 (6, 22)
Emilia Romagna, Italy	8 (3, 21)	14 (5, 31)	31 (17, 54)	7 (4, 18)
Finland	7 (3, 21)	19 (8, 41)	23 (11, 50)	12 (4, 26)
Wales	6 (2, 18)	11 (3, 28)	15 (5, 36)	6 (2, 16)
Thames Valley, UK	7 (3, 21)	12 (5, 24)	21 (10, 39)	9 (4, 19)
Wessex, UK	9 (3, 25)	12 (6, 26)	24 (13, 45)	9 (4, 21)
East Midlands & South Yorkshire, UK	7 (3, 25)	15 (6, 35)	31 (17, 62)	9 (4, 21)
Valencian Region, Spain	9 (3, 23)	17 (7, 31)	18 (8, 32)	15 (5, 29)
Overall median and 95% CI of median	7 (7, 8)	14 (12, 17)	23 (19, 28)	9 (8, 11)

Abbreviations: CHD, congenital heart disease; UK, United Kingdom.

Overall, a slightly higher percentage of children with all anomalies underwent surgery than children with Down syndrome (pooled estimate: 38.3% vs. 33.4%, Table 3). However, there were more marked differences in individual regions; for example, in Wessex, 48.9% of children with all anomalies required surgery compared to 31.4% with Down syndrome.

**TABLE 3:** Number of children who underwent surgery in the first year of life for those with Down syndrome and all anomalies.

Registry	Children with all anomalies requiring surgery			Children with Down syndrome requiring surgery		
	Total children with all anomalies	Total children needing surgery	% (95% CI)	Total children with down syndrome	Total children with Down syndrome needing surgery	% (95% CI)
Tuscany, Italy	4225	1315	31.1 (29.7, 32.5)	148	53	35.8 (28.1, 44.1)
Emilia Romagna, Italy	5381	1998	37.1 (35.8, 38.4)	206	62	30.1 (23.9, 36.7)
Finland	38,324	10,391	27.1 (26.7, 27.6)	1205	421	34.9 (32.2, 37.8)
Wales	17,448	7417	42.5 (41.8, 43.2)	562	246	43.8 (39.7, 48.0)
Thames Valley, UK	3845	1741	45.3 (43.7, 46.9)	262	67	25.6 (20.4, 31.3)
Wessex, UK	4320	2111	48.9 (47.3, 50.4)	334	105	31.4 (26.5, 36.7)
EMSY, UK	11,278	4745	42.1 (41.1, 43.0)	682	245	35.9 (32.3, 39.7)
Valencian Region, Spain	4260	1365	32.0 (30.6, 33.5)	155	43	27.7 (20.9, 35.5)
Total	89,081	31,083	38.3 (32.0, 44.5)	3554	1242	33.4 (29.6, 37.3)

Abbreviation: EMSY, East Midlands and South Yorkshire.

### 3.2 Need for More Advanced Care

**FIGURE 2:**
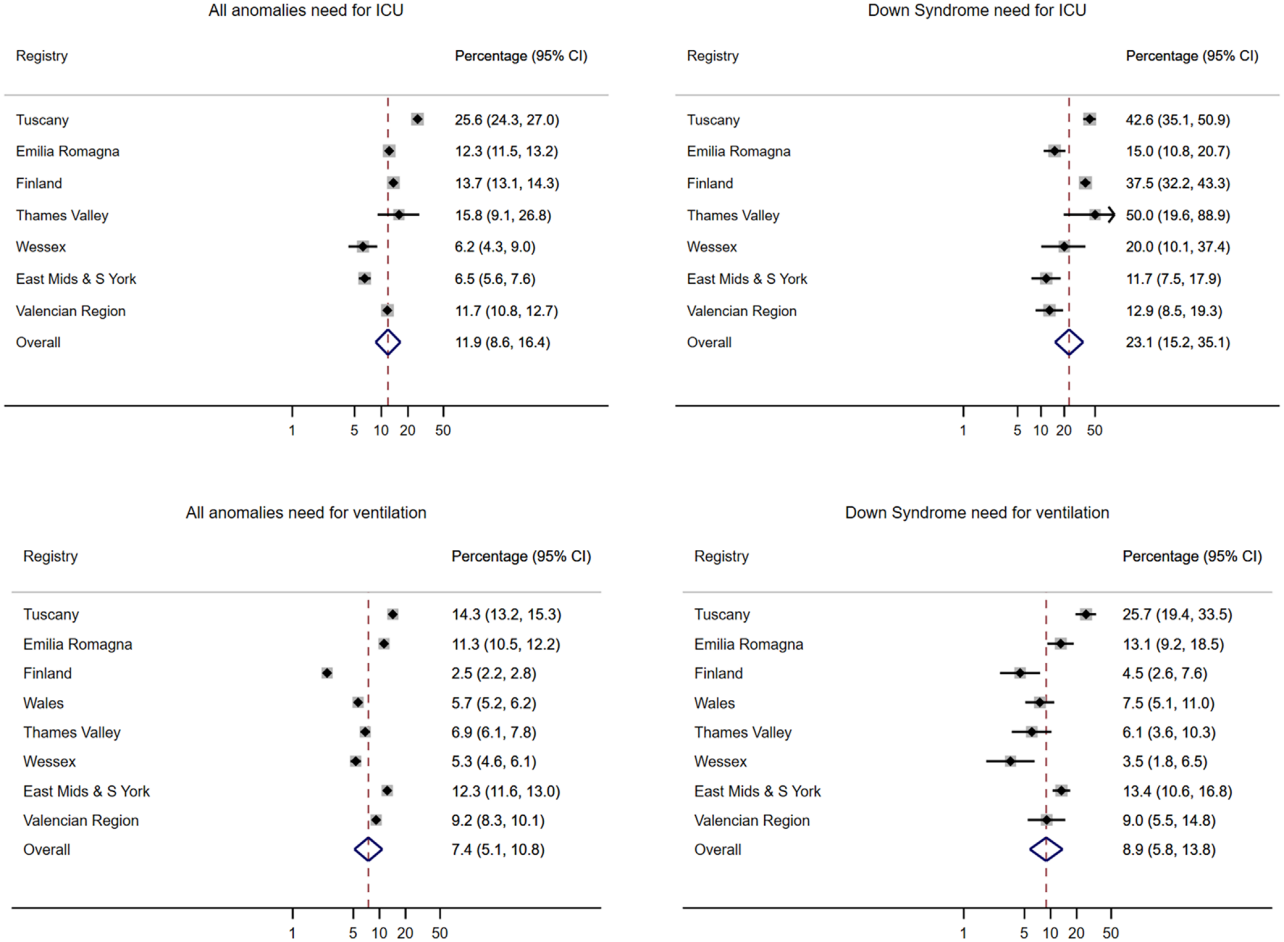
Need for admission to intensive care or required ventilation in the first year of life.

Finally, we considered the children's need for admission to intensive care and need for ventilation in the first year of life. The number of children included in the intensive care analysis was 29,383 (all anomalies) and 995 (Down syndrome), and in the ventilation analysis, it was 41,507 (all anomalies) and 1752 (Down syndrome). In this subset of children, we observed that the overall need for admission to intensive care was much higher among those children with Down syndrome versus those with all anomalies (25% vs. 13%, Figure 2) and this was true across all regions. Both groups of children had similar levels of needing ventilation (9% vs. 10%, Figure 2) overall although this varied by region, for example, in Tuscany, this was 14% of children with all anomalies and 26% of children with Down syndrome.

### 3.3 Comment

#### 3.3.1 Principal Findings

Our study examined the healthcare needs of 3554 children living with Down syndrome over the first year of life across eight European regions. Survival for these children in the first year was 95.4% overall, and consistently above 93% across all regions. We identified high levels of hospital admissions outside of the birth admission, and need for surgery varied across regions. While we have limited data, our findings suggest that children with Down syndrome were potentially admitted to intensive care units with less severe illness, indicated by higher admission rates but similar levels of ventilation need compared to children with all anomalies.

#### 3.3.2 Strengths of the Study

The major strength of our study is the population‐based approach, which does not rely on records from individual hospitals and can result in biases. Pooling standardised data from multiple registries across several European countries allows for more accurate quantification of risks of specific outcomes for children living with Down syndrome. The included registries provide standardised data from high‐quality population‐based registers who are all members of EUROCAT and follow a consistent approach to coding. Each register includes cases of congenital anomalies occurring in live births, stillbirths, termination of pregnancy for foetal anomaly and late miscarriage. This study relied on the successful linkage of children with their healthcare data, which has been shown in previous EUROlinkCAT studies to be high (> 95%) and similar for children with congenital anomalies and those without in the wider project [4]. Pooling data from a number of registries across several European regions have enabled the association of additional anomalies with Down syndrome to be explored.

#### 3.3.3 Limitations of the Data

We were unable to undertake any analysis on an individual level and were restricted to the analyses we were able to perform on aggregated data. We were unable to account for any potential confounders (e.g., preterm birth) due to not having child‐level data. However, a strength of our work is that we used appropriate statistical approaches, including random‐effects meta‐analysis, to pool results. We could not compare children with Down syndrome to a group that did not include Down syndrome, as the primary aim of EUROlinkCAT was to describe outcomes for children with specific congenital anomalies as a whole (comprising children with isolated anomalies as well as those with associated anomalies). However, because Down syndrome makes up a small percentage of all congenital anomalies, the comparison is approximately that of two distinct groups (children with Down syndrome make up ~4% of the overall congenital anomaly group). We also had a substantially reduced sample size when considering need for intensive care and ventilation as data on these outcomes were only available for a restricted number of years for several registries due to changes in local data collection procedures over time. We could not consider the coexistence of other anomalies and we were unable to explore the reasons for the need for intensive care admission or need for ventilation. This could relate to their heart condition (for those who have them) or being at increased risk of other complications such as respiratory conditions.

#### 3.3.4 Interpretation

While overall survival was around 95%, this was lower when considering children with additional structural anomalies. For those children who also had CHD and a gastrointestinal anomaly, survival was 89%. Other research has shown that the probability of survival is substantially lower in children with Down syndrome and CHD compared to matched controls from the general population and compared to children with only CHD [20]. This demonstrates the added complexity of living with multiple lifelong health conditions as a child and into adulthood, the numbers of which have been increasing over time [21].

In all children with Down syndrome, we observed an overall hospital admission rate of 88%, similar to that seen in previous research from Scotland which observed a hospitalisation rate of > 90% which had increased slightly over time [10]. The high hospitalisation rate we observed was lower for those children with Down syndrome without CHD, but it still remained above 80%, consistently higher than for all children with congenital anomalies.

The median length of stay in hospital in the first year of life was consistently higher for children with Down syndrome compared to those children with all anomalies across all regions. The median total length of hospital stay ranged from 3 days longer (Wessex) to 12 days (Finland and Tuscany). This is similar to other research which has shown that children with Down syndrome are admitted at a younger age and have longer inpatient stays than children without Down syndrome [11]. We were able to compare children with Down syndrome with CHD and those without CHD and this demonstrated that much longer lengths of stay were seen in children with Down syndrome and CHD (23 days vs. 9 days).

Variations in surgery across regions may have resulted from variations in termination rates due to differences in prenatal screening practice. For example, first‐trimester ultrasound screening is more likely to pick up foetuses with CHD which may lead to a termination of the pregnancy. Therefore, if such screening occurs, the proportion of live births with CHD may be lower than in regions without this screening.

Our finding that children with Down syndrome were potentially admitted to intensive care units with less severe illness may reflect a cautious approach to their care such as following surgery where other children might be cared for in lower‐level environments, children with Down syndrome may be admitted to intensive care. This reflects previous work where children with Down syndrome had potentially higher than expected organ support given their disease severity [12]. This is important for families and healthcare professionals to explore further as admission to intensive care is not without risks including being traumatic for families [22, 23] when other settings may have been as or more appropriate to provide care. However, these findings should be interpreted with caution as data quality is likely to be poorer for these variables and collection may vary across regions. We also do not know the reasons for the child's admission to intensive care.

## 4 Conclusions

There is a high need for hospital care for children born with Down syndrome in the first year of life, but we found indications that this care may not always be in the most appropriate location. It is important that information about the care pathway from antenatal diagnosis to 1 year of age is shared with families. Future work should continue to explore the long‐term prognosis for children with Down syndrome to ensure their care needs are met.

## Author Contributions

J.K.M., J.R., M.L. and D.T. obtained funding for the original EUROlinkCAT project. S.E.S. completed the analysis under the supervision of J.R. and J.K.M. S.S. wrote the first draft of the paper. All authors revised the paper providing critique and intellectual contribution.

## Ethics Statement

All registries obtained ethical and other permissions for the data linkage according to their national legislations. The University of Ulster obtained ethics permission for the Central Results Repository on 15 September 2017 (Institute of Nursing and Health Research Ethics Filter Committee, Number FCNUR‐17‐000).

## Conflicts of Interest

The authors declare no conflicts of interest.

## Supporting information


Table S1.

## Data Availability

The data in the EUROlinkCAT project are sensitive as they relate to children with congenital anomalies, many of which are rare. The study team had access to aggregate data only from each region, that is, the linked patient‐level data remained in the local region. We will make all our documentation available and encourage any interested parties to apply to the EUROlinkCAT management team to assist them in obtaining approval from the data providers in each region/country to use the aggregated data for an approved study.
