# Supplementary material for: The Healthcare Needs of Children With Down Syndrome in the First Year of Life: An Analysis of the EUROlinkCAT Data Linkage Study
Source: Paediatr Perinat Epidemiol. 2025 Feb 6;39(5):394–401. doi: 10.1111/ppe.13176 (PMC12308622; doi:10.1111/ppe.13176)
Supplement: Supplementary file 1 — Table S1. [file PPE-39-394-s001.docx]

**Table S1:** Overall one-year survival of children with Down syndrome and other associated anomalies

|  | Tuscany, Italy | Emilia Romagna, Italy | Finland | Wales | Thames Valley, UK | Wessex, UK | EMSY, UK | Valencian Region, Spain | Overall |
| --- | --- | --- | --- | --- | --- | --- | --- | --- | --- |
|  | %  (95% CI) | %  (95% CI) | %  (95% CI) | %  (95% CI) | %  (95% CI) | %  (95% CI) | %  (95% CI) | %  (95% CI) | Pooled %  (95% CI) |
| All Down syndrome | 95.5  (90.7, 97.8) | 97.3  (94.0, 98.8) | 95.6  (94.3, 96.5) | 93.7  (91.4, 95.4) | 97.2  (94.3, 98.7) | 96.3  (93.6, 97.9) | 92.7  (90.4, 94.4) | 95.5  (92.2, 97.4) | 95.4  (94.3, 96.5) |
| Down syndrome with CHD and gastrointestinal | -* | -* | 90.9  (83.3, 95.2) | 85.0  (69.6, 92.7) | 92.3  (56.6, 98.9) | 92.3  (56.6, 98.9) | 80.0  (56.6, 98.9) | 81.8  (44.7, 95.1) | 88.9  (84.2, 93.6) |
| Down syndrome with CHD and no gastrointestinal | 90.2  (78.0, 95.8) | 92.5  (84.1, 96.6) | 95.2  (93.4, 96.6) | 93.7  (90.5, 95.9) | 98.4  (89.3, 99.8) | 93.1  (84.1, 97.0) | 89.6  (84.6, 93.0) | 95.5  (90.8, 97.8) | 94.1  (92.5, 95.8) |
| Down syndrome with gastrointestinal and no CHD | 88.9  (43.3, 98.4) | -* | 96.1  (85.2, 99.0) | 94.1  (65.0, 99.1) | -* | -* | 95.7  (72.9, 99.4) | -* | 95.5  (89.9, 100.0) |
| Down syndrome with no CHD and no gastrointestinal | 98.9  (92.4, 99.8) | -* | 97.0  (95.0, 98.2) | 95.2  (91.2, 97.4) | 97.1  (93.2, 98.8) | 97.3  (94.2, 98.8) | 94.9  (92.2, 96.6) | 96.8  (90.5, 99.0) | 96.6  (90.5, 99.0) |

*Due to too few births, not estimated and excluded from pooled estimates

CHD: congenital heart disease; EMSY: East Midlands and South Yorkshire; UK: United Kingdom
